# Supplementary material for: Complexome profiling of the Chlamydomonas psb28 mutant reveals TEF5 as an early PSII assembly factor
Source: Plant Cell. 2025 Mar 17;37(6):koaf055. doi: 10.1093/plcell/koaf055 (PMC12164590; doi:10.1093/plcell/koaf055)
Supplement: koaf055_Supplementary_Data [file koaf055_supplementary_data.zip › psb28_interactive.zip!/plots/intensity/qualityPlots/peptides/LHCA3_Cre11.g467573.t1.1.html]

### Cre11.g467573.t1.1

Trivial name: LHCA3  
SeqCoverage [median]: 43.80  
Mass: 28.89 kDa  
  
 

| Sequence | MissedCleavage | Mass [Da] | wtIntensity | muIntensity | Score |
| --- | --- | --- | --- | --- | --- |
| AEEKSIAKVDR | 2 | 1244.67 | 406.34 | 3933.98 | 101.89 |
| GPFQNLVEHLADPVNNNILTNFGK | 0 | 2650.35 | 180734.65 | 77809.18 | 283.55 |
| GSGDAAYPGGPFFNLFNLGK | 0 | 2027.97 | 312177.39 | 117014.88 | 171.62 |
| LAMLAMLGYGAQAVMTGK | 0 | 1824.93 | 417595.80 | 745056.19 | 213.94 |
| LAMLAMLGYGAQAVMTGKGPFQNLVEHLADPVNNNILTNFGK | 1 | 4457.27 | 11609.34 | 0.00 | 66.38 |
| LQDFRYPGSMGQQYFLGLEAIFK | 1 | 2707.35 | 27980.56 | 0.00 | 116.94 |
| RLQDFR | 1 | 833.45 | 100404.04 | 117037.11 | 144.29 |
| SIAKVDR | 1 | 787.46 | 0.00 | 8743.37 | 126.52 |
| TEAAMKELK | 1 | 1019.53 | 112058.93 | 94739.72 | 174.02 |
| WLQYSEVIHAR | 0 | 1400.72 | 925808.31 | 798348.87 | 185.18 |
| YPGSMGQQYFLGLEAIFK | 0 | 2048.01 | 119092.48 | 0.00 | 169.54 |
|  |  |  | 2207867.8 | 1962683.3 |  |
